# Comparison of spinal anesthesia and general anesthesia in inguinal hernia repair in adult: a systematic review and meta-analysis

**DOI:** 10.1186/s12871-020-00980-5

**Published:** 2020-03-10

**Authors:** Lin Li, Yi Pang, Yongchao Wang, Qi Li, Xiangchao Meng

**Affiliations:** Department of Thyroid, Breast, Hernia Surgery, Tianjin the third Central Hospital, NO.83, Jintang Road, Tianjin, 300170 China

**Keywords:** Inguinal hernia repair, Spinal anesthesia, General anesthesia, Meta-analysis

## Abstract

**Background:**

Inguinal hernia repair is one of the most commonly performed surgical procedures. To date, there is no consensus on which anesthesia should be used. The objective of this meta-analysis was to assess the efficacy of spinal anesthesia (SA) vs. general anesthesia (GA) in inguinal hernia repair in adults.

**Methods:**

Eligible studies were identified before January 2020 from PubMed, Embase, ScienceDirect, Cochrane Library, Scopus database as well as reference lists. Outcomes included surgery time, the time in the operation room, the length of hospital stay, pain scores, patient satisfaction, and postoperative complications. Subgroup analysis based on surgical approaches was conducted.

**Results:**

Six randomized controlled trials (RCT) and five cohort studies were included. A total of 2593 patients were analyzed. Compared to GA, SA was associated with a longer surgery time (weighted mean difference [WMD]: − 3.28, 95%confident interval [CI]: − 5.76, − 0.81), particularly in laparoscopic repair. Postoperative pain at 4 h and 12 h were in favor of SA following either open or laparoscopic repairs (standard mean difference [SMD]: 1.58; 95%CI: 0.55, 2.61, SMD: 0.99, 95%CI: 0.37, 1.60, respectively); and considering borderline significance, patients receiving SA might be more satisfied with the anesthesia they used for herniorrhaphy (SMD: -0.32, 95%CI: − 0.70, 0.06). Some major complications of scrotal edema, seroma, wound infection, recurrence, shoulder pain were comparable between the two groups. However, patients receiving SA had an increased risk of postoperative urinary retention and headache when compared with GA (relative ratio [RR]: 0.44, 95% CI: 0.23, 0.86, RR: 0.33, 95% CI: 0.12, 0.92, respectively). There was a tendency that the incidence of postoperative nausea and vomiting was lower in SA than GA (RR: 2.12, 95%CI: 0.95, 4.73), especially in open herniorrhaphy.

**Conclusions:**

SA can be another good choice for pain relief no matter in open or laparoscopic hernia repairs, but it can’t be confirmed that SA is better than GA.

## Background

Inguinal hernia repair is one of the most commonly performed surgical procedures every year [1]. Patients always expect to undergo this operation with little anesthetic risk, minimal discomfort, and early recovery and discharge home. To date, there is no consensus on which anesthesia should be used. The choice of anesthetic techniques ranges from local infiltration to regional block to general endotracheal. Local anesthesia (LA) is more frequently used in specialist hernia centers, however, infiltration is painful and 85% of patients experience pain intraoperatively [2]. The most commonly used regional anesthesia technique is spinal anesthetic (SA), which has the advantage of avoiding paralytic agents and endotracheal intubation [3]. General anesthesia (GA) is most preferred by patients because of anxiety and fear of surgery, with a frequency of 60–70% [4]. Many studies have attempted to explore the benefits among the three anesthetic techniques for inguinal hernia repair. However, to date, no pooled analyses of the results focusing on the comparison between SA and GA in adults have surfaced. The purpose of this meta-analysis was to assess the efficacy of SA vs. GA in inguinal hernia repair in adults, in terms of surgery time, the time in operation room, hospital stay, pain scores, patient satisfaction, and major postoperative complications.

## Methods

This meta-analysis was carried out according to the guidelines of the Preferred Reporting Items for Systematic Reviews and Meta-Analysis statement [5].

### Search strategy

The primary search of electronic databases was conducted in PubMed, Embase, ScienceDirect, Cochrane Library, and Scopus database before January 2020. Supplemental identification was conducted by cross-checking of reference lists. Combinations of search terms ‘spinal anesthesia’, ‘general anesthesia’, and ‘inguinal hernia’ were used. Two reviewers independently checked the titles and abstracts of potentially relevant studies. Studies were excluded due to duplication, non-related topics and other article types (review, case report, etc.). Differences between reviewers were resolved by discussion until agreement was reached.

### Study inclusion criteria

Only randomized controlled trails (RCTs) or cohort studies that compared spinal anesthesia with general anesthesia used in inguinal hernia repair in adults could be included. The language reported on need to be English, but region and publication date were free from limitation. Study results should cover intraoperative or postoperative outcome measures. However, studies that included a double anesthetic procedure to the same group of patients were eliminated from this analysis.

### Data extraction

Data from eligible records were reviewed and extracted into an Excel spreadsheet. Our measurements encompass surgery time, the time in the operation room, the length of hospital stay, pain score, patient satisfaction, and postoperative complications. Outcomes of complications assessed in at least three papers were considered for meta-analysis. For the study of Sunamak et al. assessing open and laparoscopic total extraperitoneal repairs under GA and SA, we extracted two sets of data for meta-analysis, namely data for open repair and data for laparoscopic repair.

We defined the surgery time as the duration between beginning of the skin incision and skin closure; the time in the operating room as the period from the beginning of anesthesia to discharge from the operating room. Complications include scrotal edema, seroma, wound infection, recurrence, shoulder pain, urinary retention, headache, and postoperative nausea and vomiting (PONV).

### Quality assessment

Quality of each included RCT was evaluated using the Cochrane Collaboration tool for assessing the risk of bias [6]. The method contains seven domains, namely, random sequence generation, allocation concealment, blinding of participants and personnel, blinding of outcome assessment, incomplete outcome data, selective reporting, and other bias. For each domain, RCTs were assessed to be high (red), unclear (yellow), or low (green) in risk of bias. As to the quality assessment of cohort studies, the Newcastle-Ottawa Scale was used [7]. The tool includes three domains: the selection of the study groups; the comparability of the groups; and the ascertainment of either the exposure or outcome of interest for cohort studies. For each evaluation, a ‘star system’ was applied to score from 0 star to 9 stars.

### Data analysis

We conducted all statistical analysis using Stata software version 15.0 (Stata Corporation, College Station, TX, USA) software. Pooled relative ratios (RRs) and 95% confidence intervals (CIs) were calculated for postoperative complications. Weighted mean differences (WMDs) and 95% CIs were calculated for surgery time, operation time, and the length of hospital stay; standard mean differences (SMDs) and 95% CIs were calculated for pain scores and patient satisfaction. Heterogeneity across the studies was estimated by the I^2^ statistics. I^2^ > 50% was defined as significant heterogeneity. In case of I^2^ > 50%, a random effects model was used, otherwise, a fixed effects model was preferred. Sensitivity analysis was performed when significant heterogeneity was found. Sensitivity analysis was conducted by removing one study at a time to disclose if one particular study could affect the overall result. Subgroup analyses of laparoscopic and open techniques were also conducted. Pooled outcomes were presented in forest plots and considered as statistically significant if *P* value < 0.05.

## Results

### Study screening

**Fig. 1:**
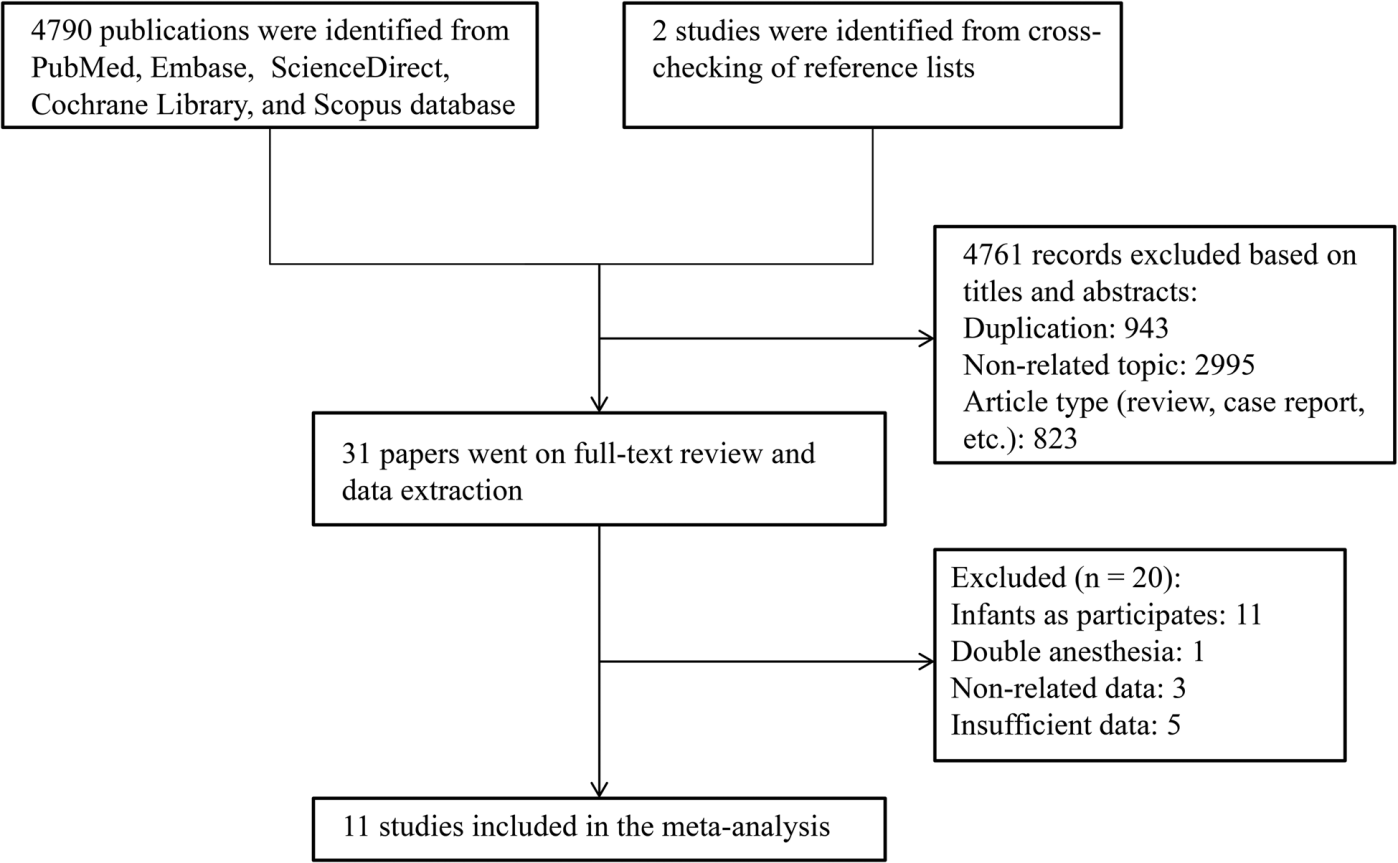
Flow diagram describing the article search and inclusion in meta-analysis

The initial search of electronic databases produced 4790 studies. Two supplemental records were identified through checking the references of above mentioned studies. Further screening removed 4761 articles due to duplication, non-related topics and other article types (reviews, case reports, etc). Thirty one potentially eligible studies remained and went on a full-text review. We finally included 11 studies that matched the aforementioned criteria for this meta-analysis. The flow diagram describing the article search was shown in Fig. 1.

### Study characteristics

Six RCTs and five cohort studies were included [3, 8–17]. A total of 2593 patients were recruited into this analysis. The patient age was comparable in each selected study, and the inguinal hernia included in all studies were uncomplicated hernia. Open inguinal hernia repair was performed in six studies, and laparoscopic surgical techniques were used in six. Study characteristics were summarized in Table 1.

**Table 1:** Study characteristics

Study	Design	Group	Total N	Age	N	Inclusion	Exclusion	Repair used
Burney RE 2004 [4]	RCT	SA	33	≥18 years	15	Unilateral hernia	Recurrent or bilateral hernia	Open inguinal repair
		GA		≥ 18 years	18			
Donmez T 2016 [8]	RCT	SA	50	37.16 ± 10.85	25	Uncomplicated hernia	Complicated inguinal hernia (irreducible, obstructed, or strangulated); Recurrent hernias	TEP
		GA		35.36 ± 11.40	25			
Ismail M 2009 [10]	Cohort study	SA	652	46.1 ± 14.1	636	Reducible inguinal hernia	Obstructed and strangulated hernias, pediatric hernias, and other hernias, such as ventral hernias	TEP
		GA		43.3 ± 15.6	16			
Ozgün H 2002 [15]	RCT	SA	50	51.4 ± 15.1	25	Unilateral, reducible, direct or indirecthernia; types II and III according to the Nyhus classication	Scrotal, sliding, recurrent hernias	Open inguinal repair
		GA		46.9 ± 19.8	25			
Pere 2016 [16]	RCT	SA	100	51 ± 15	49	Unilateral	Not reported	Open inguinal repair
		GA		54 ± 15	51			
Sarakatsianou C 2017 [11]	RCT	SA	70	58.85 ± 13.54	34	Non-high risk; primary, unilateralinguinal hernia	Non-reducible/obstructed hernias, bilateral hernias, big scrotal hernias	TAPP
		GA		57.64 ± 15.77	36			
Sinha R 2008 [9]	Cohort study	SA	529	32.2	480	Unilateral or bilateral, direct or indirect, recurrent inguinal hernia	Obstructed and strangulatedinguinal hernia	TEP
		GA		33.7	49			
Sunamak 2018 (1) [12]	Cohort study	SA	207	31.8 ± 10.9	96	Unilateral hernia	Recurrent hernias, strangulated, incarcerated, or bilateral hernia	TEP
		GA		39.9 ± 16.2	111			
Sunamak 2018 (2) [12]	Cohort study	SA	233	38.1 ± 16.8	116	Unilateral hernia	Recurrent hernias, strangulated, incarcerated, or bilateral hernia	Open inguinal repair
		GA		39.1 ± 16.5	117			
Symeonidis 2013	Cohort study	SA	75	56.04 ± 13.44	50	Unilateral	Scrotal, recurrent, bilateral, strangulated, or incarcerated hernias	Open inguinal repair
		GA		61.28 ± 11.42	25			
Urbach 1964 [13]	RCT	SA	514	48 (17–71)	236	Unilateral or bilateral inguinal hernia	Not reported	Open inguinal repair
		GA		43 (18–75)	278			
Yildirim D 2017 [14]	Cohort study	SA	80	35.0 ± 11.3	40	Direct or indirect hernia	Strangled, bilateral, hernia, recurrent hernia	TEP
		GA		36.4 ± 10.0	40			

*RCT* Randomized controlled trail, *SA* Spinal anesthesia, *GA* General anesthesia, *TEP* Laparoscopic total extraperitoneal hernia repair, *TAPP* Laparoscopic transabdominal preperitoneal inguinal hernia repair

### Study quality

**Fig. 2:**
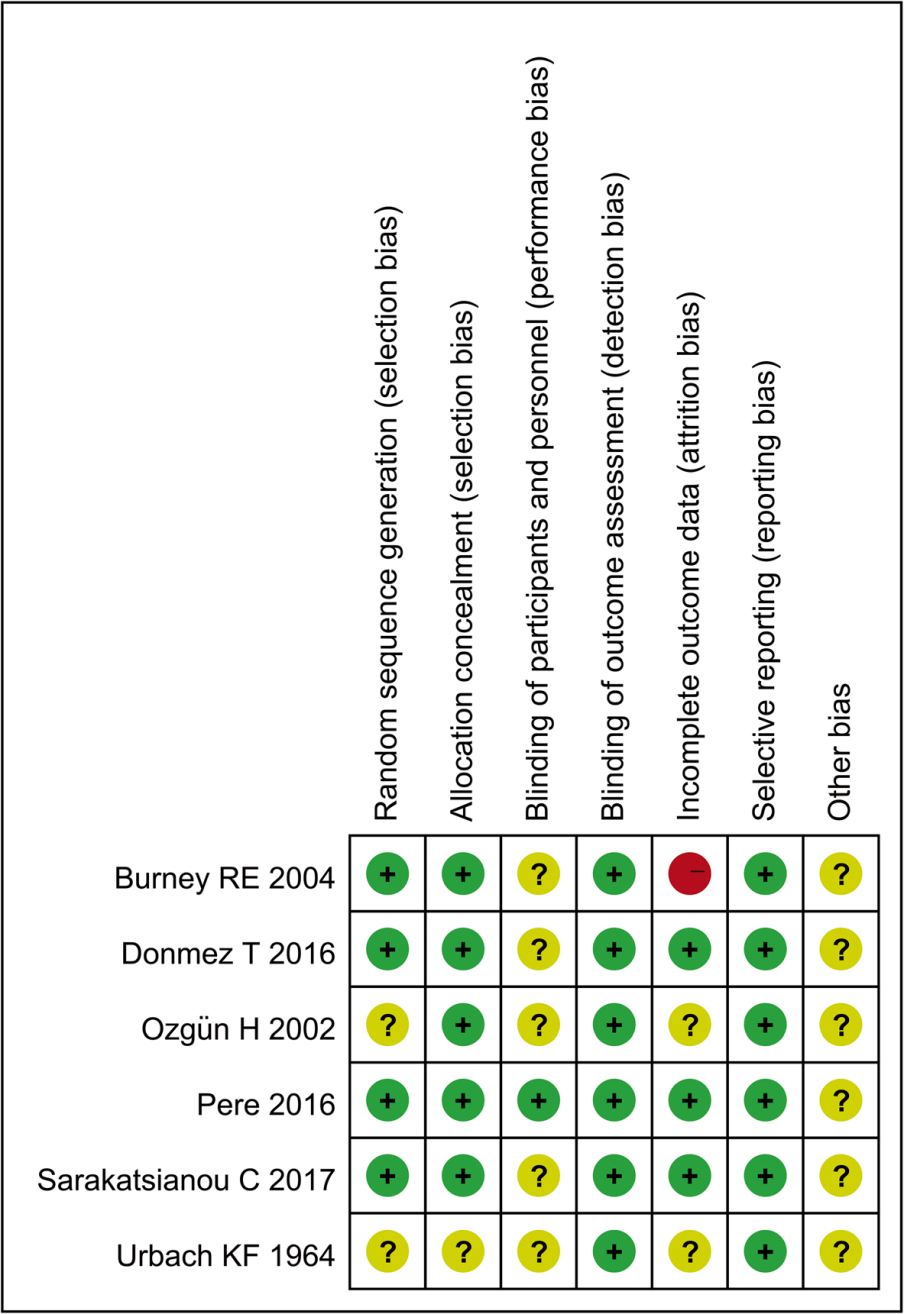
Risk of bias summary of five included RCTs

The evaluation for quality of each study was shown in Table 2 and Fig. 2. Both randomization and the method of sequence generation were mentioned in three RCTs [3, 8, 11], whilst allocation concealment was described in four RCTs [3, 8, 11, 15]. All the five RCTs failed to provide adequate information of blinding, so they might involve an unclear risk of bias. And all the RCTs analyzed were judged to have low or unclear risk of incomplete outcome data, selective outcome reporting and other potential source of bias. On the other hand, four cohort studies satisfied all criteria and scored 9 stars [10, 12, 14]. Only one cohort study scored 8 stars owing to inadequacy of follow up [9].

**Table 2:** Quality assessment for each included cohort study

Item	Ismail M 2009 [10]	Sinha R 2008 [9]	Sunamak 2018 [12]	Symeonidis 2013	Yildirim D 2017 [14]
Item 1: The selection of the study groups					
Representativeness of the exposed cohort	☆	☆	☆	☆	☆
Selection of the non exposed cohort	☆	☆	☆	☆	☆
Ascertainment of exposure	☆	☆	☆	☆	☆
Demonstration that outcome of interest was not present at start of study	☆	☆	☆	☆	☆
Item 2: The comparability of the groups	☆☆	☆☆	☆☆	☆☆	☆☆
Item 3: The ascertainment of either the exposure or outcome of interest for cohort studies					
Assessment of outcome	☆	☆	☆	☆	☆
follow-up long enough for outcomes to occur	☆	☆	☆	☆	☆
Adequacy of follow up of cohorts	☆		☆	☆	☆

☆: one ☆ means one score; each domain of Item 1 and 3 worth one ☆, Item 2 worth two ☆

### Outcomes

**Fig. 3:**
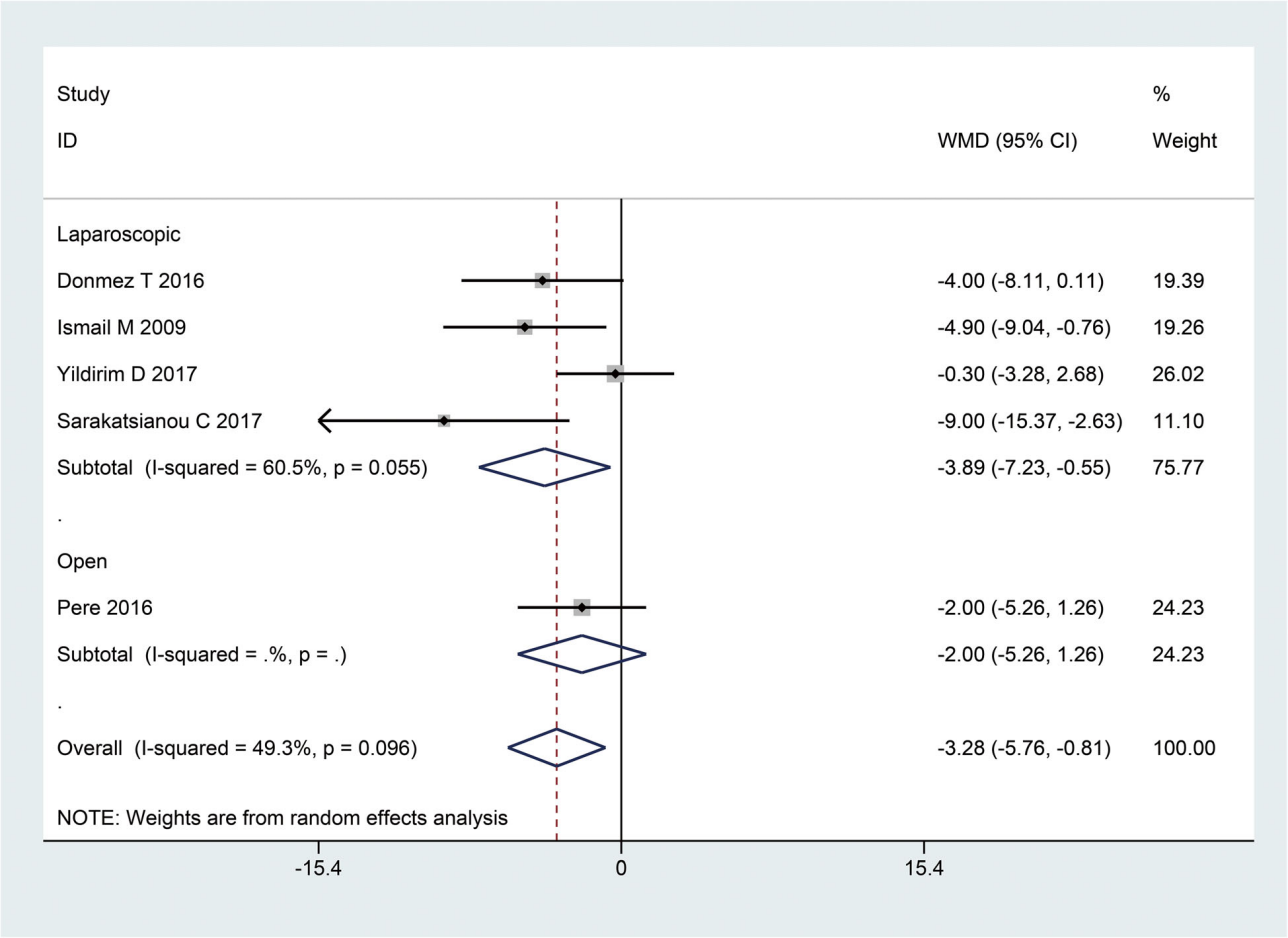
The surgery time when GA and SA compared

Five studies evaluated the surgery time and the pooled WMD was − 3.28 (95%CI: − 5.76, − 0.81; I^2^ = 49.3%) in favor of GA (*P* = 0.01); however, subgroup analysis suggested the statistical significance to only remain in laparoscopic repair group (WMD: -3.89, 95%CI: − 7.23, − 0.55, *P* = 0.02) (Fig. 3).

**Fig. 4:**
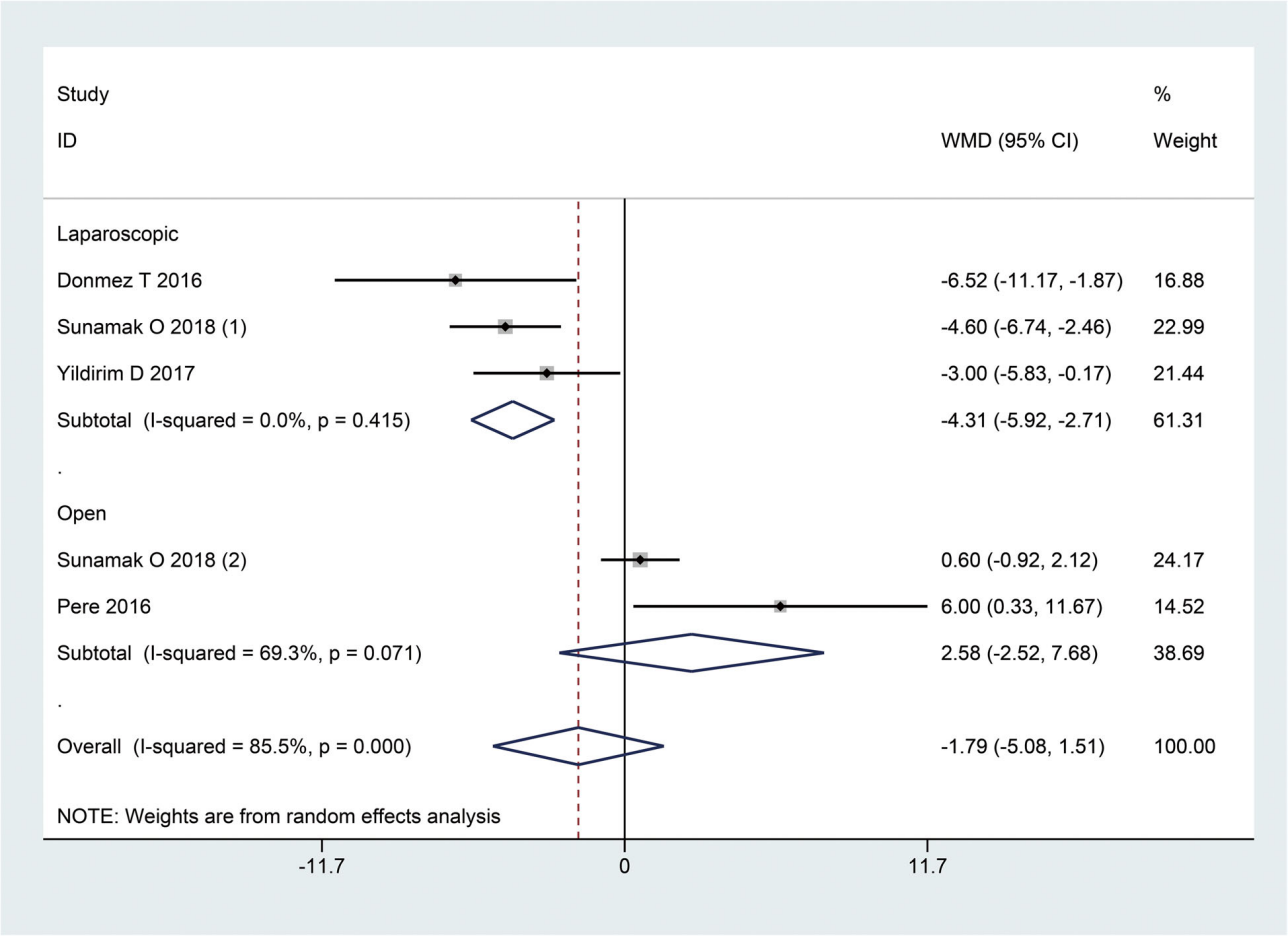
The time in operation room when GA and SA compared

Data synthesis of operating time from five cohorts generated a WMD of − 1.79 (95%CI: − 5.08, 1.51; I^2^ = 85.5%), indicating the time in the operating room was comparable between SA and GA groups (*P* = 0.29). Sensitivity analysis did not change the result or heterogeneity. Interestingly, by subgroup analysis we found SA group had significantly longer operation time than GA group following laparoscopic repair (WMD: -4.31, 95%CI: − 5.92, − 2.71, *P* < 0.01) (Fig. 4).

**Fig. 5:**
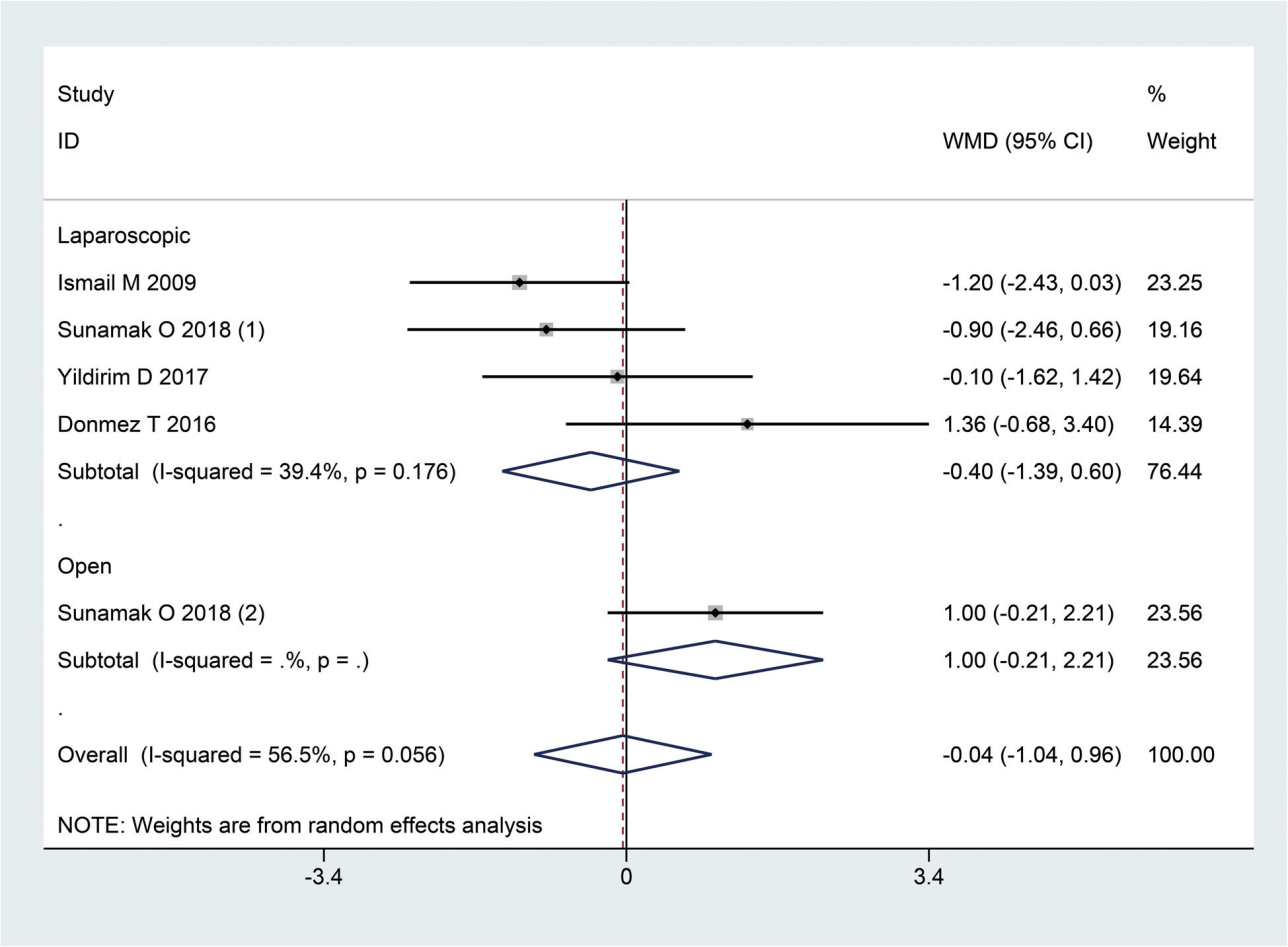
The hospital stay when GA and SA compared

There was no significant difference in the length of hospital stay between patients under SA and GA (WMD: -0.04, 95%CI: − 1.04, 0.96, I^2^ = 56.5%, *P* = 0.94). This comparable results remained in both subgroups of laparoscopic and open repairs (Fig. 5).

**Fig. 6:**
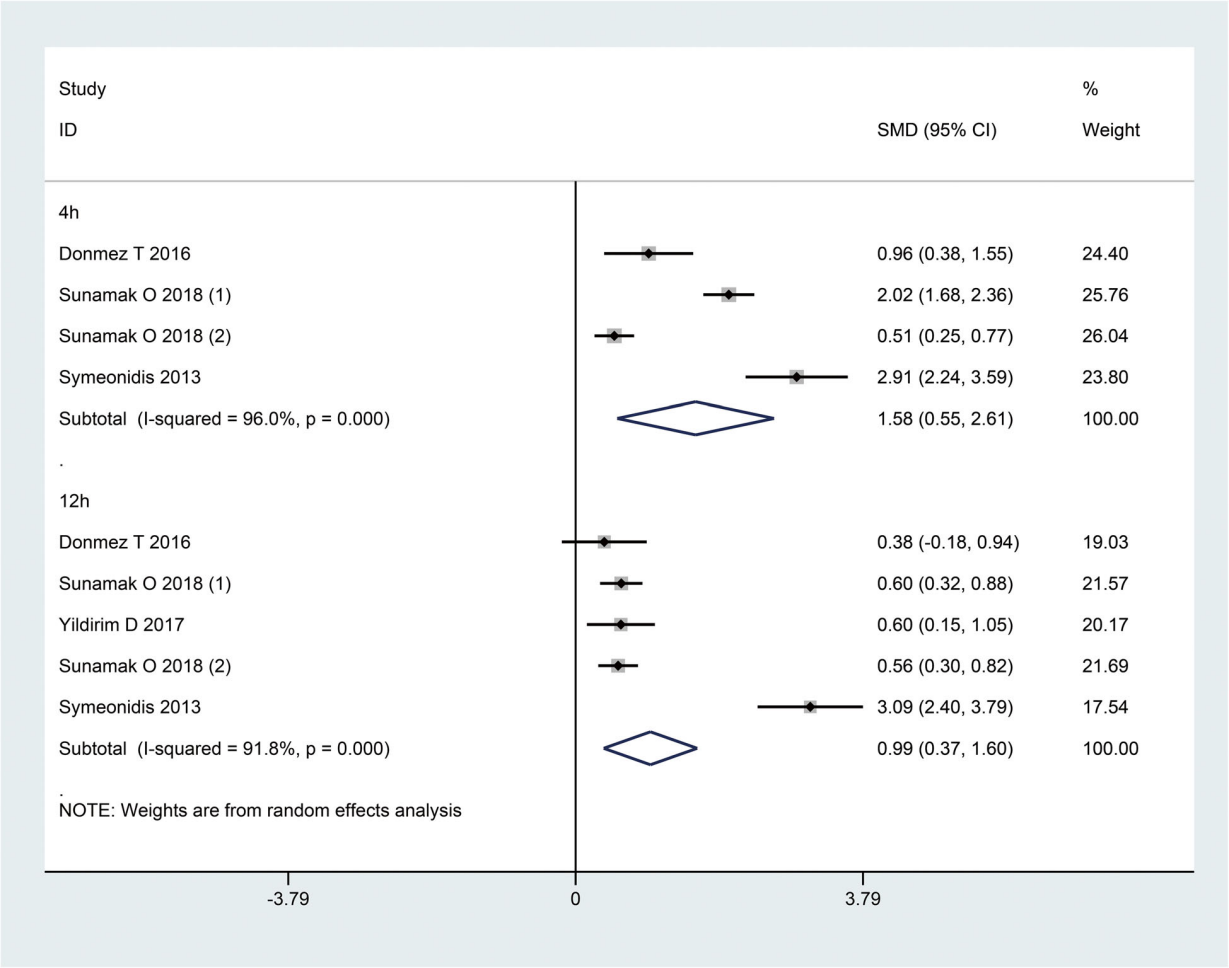
The pain scores at 4 h and 12 h after operation when GA and SA compared

We assessed pain scores at 4 h and 12 h after operation. The overall results showed that pain scores were significantly higher in patients under GA compared to SA at these two time points (SMD: 1.58, 95%CI: 0.55, 2.61, I^2^ = 96.0%, *P* < 0.01; SMD: 0.99, 95%CI: 0.37, 1.60, I^2^ = 91.8%, *P* < 0.01, respectively) (Fig. 6). In the subgroup of laparoscopic repair, statistical significance remained between the two groups. In the subgroup of open repair, there were also trends that patients with GA had higher pain scores than those with SA at these two time points, though there were no statistical significances (Table 3).

**Table 3:** Summary of subgroup analysis comparing pain scores and complications between SA and GA

Outcomes	Subgroups	SMD (95% CI)	RR (95% CI)	I^2^ value	*P* value*
Pain scores					
4 h	Laparoscopic	1.52 (0.49, 2.55)	–	89.3%	< 0.01
	open	1.69 (−0.66, 4.05)	–	97.6%	0.16
12 h	Laparoscopic	0.57 (0.35, 0.78)	–	0.0%	< 0.01
	open	1.81 (−0.68, 4.29)	–	97.8%	0.15
Complications					
Scrotal edema	Laparoscopic	–	0.64 (0.34, 1.21)	0.0%	0.17
	open	–	1.26 (0.48, 3.26)	–	0.64
Seroma	Laparoscopic	–	0.72 (0.36, 1.44)	0.0%	0.35
	open	–	1.36 (0.61, 3.02)	0.0%	0.45
Wound infection	Laparoscopic	–	0.93 (0.40, 2.17)	0.0%	0.87
	open	–	1.17 (0.49, 2.76)	0.0%	0.73
Recurrence	Laparoscopic	–	1.57 (0.44, 5.62)	0.0%	0.49
	open	–	1.21 (0.37, 3.99)	20.9%	0.75
Shoulder pain	Laparoscopic	–	1.02 (0.52, 1.98)	0.0%	0.95
	open	–	–	–	–
Urinary retention	Laparoscopic	–	0.38 (0.15, 0.95)	0.0%	0.04
	open	–	0.47 (0.18, 1.22)	71.7%	0.12
Headache	Laparoscopic	–	0.20 (0.06, 0.68)	0.0%	0.01
	open	–	0.48 (0.09, 2.47)	78.7%	0.38
PONV	Laparoscopic	–	2.15 (0.50, 9.26)	84.1%	0.31
	open	–	1.76 (1.10, 2.80)	0.0%	0.02

*: comparisons of outcomes between SA and GA; *CI* Confidence interval, *PONV* Postoperative nausea and vomiting, *RR* Relative ratio, *SMD* Standard mean difference

**Fig. 7:**
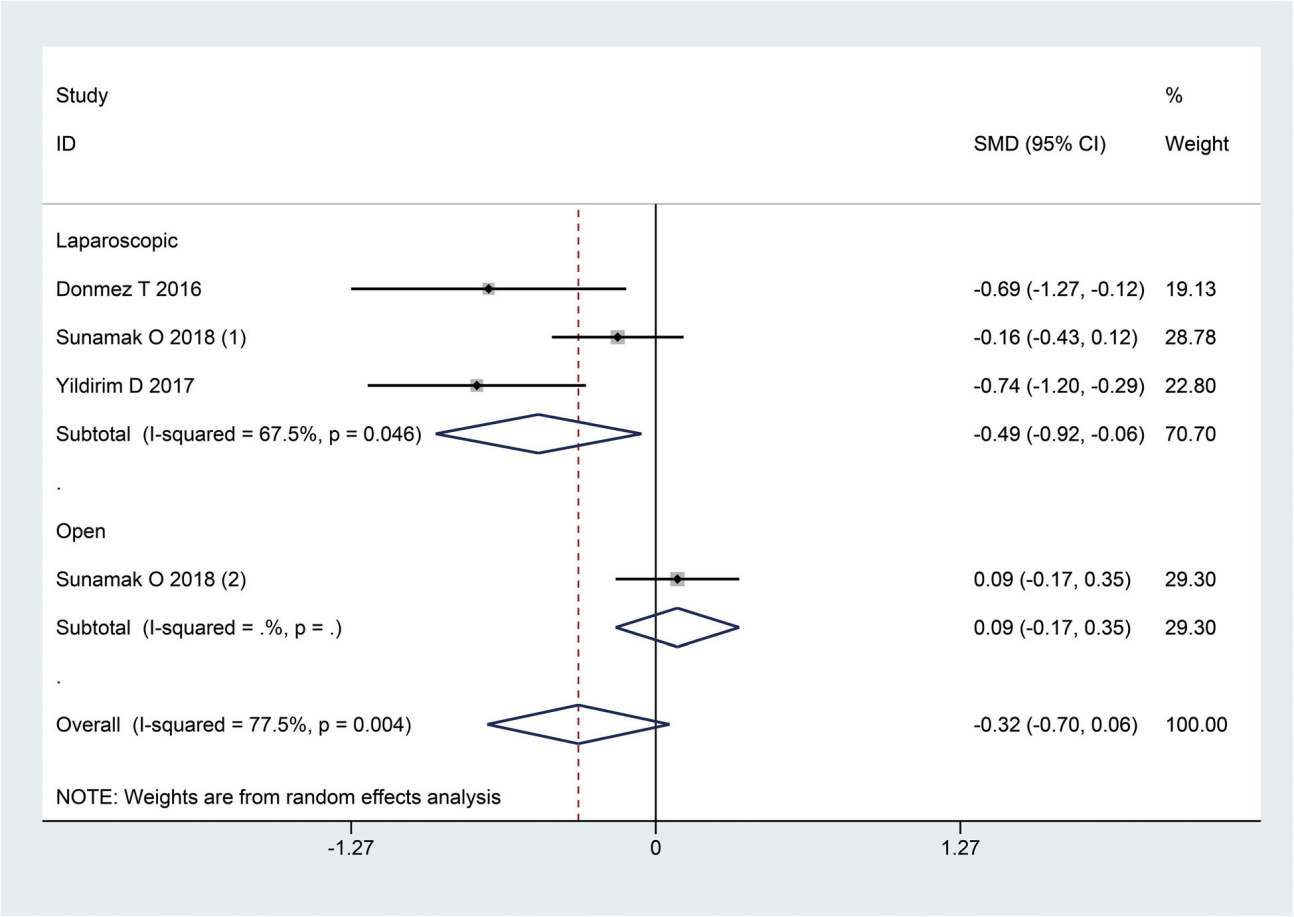
The patients satisfaction when GA and SA compared

Only four cohorts reported patient satisfaction and the pooled SMD was − 0.32 (95%CI: − 0.70, 0.06, I^2^ = 77.5%, *P* = 0.10), indicating a trend that patients receiving SA were more satisfied with the anesthesia they used for herniorrhaphy as compared to GA (Fig. 7). Subgroup analysis further revealed that the difference between the two groups regarding satisfaction was significant in favor of SA in the laparoscopic group.

**Fig. 8:**
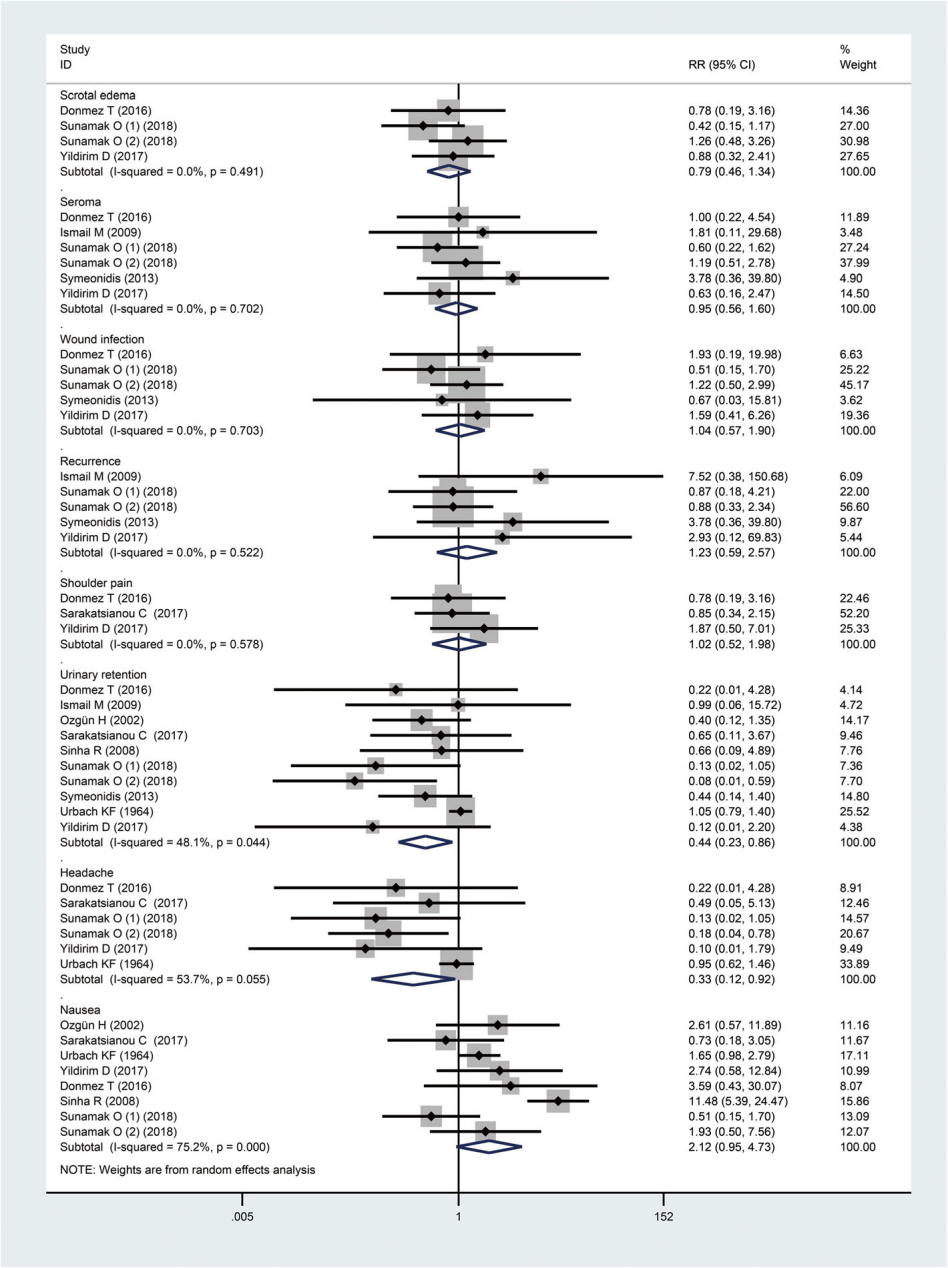
The postoperative complications when GA and SA compared

Meta-analysis of postoperative complications was shown in Fig. 8. We found the incidences of scrotal edema, seroma, wound infection, recurrence, and shoulder pain were comparable between the two groups irrespective of laparoscopic or open repairs. However, the incidences of urinary retention and headache were significantly higher in SA group than GA group (RR: 0.44, 95%CI: 0.23, 0.86, I^2^ = 48.1%, *P* = 0.02; RR: 0.33, 95%CI: 0.12, 0.92, I^2^ = 91.8%, *P* = 0.03, respectively); but we found in subgroup analysis that the significance only remained in laparoscopic repair group. For the incidence of PONV, borderline significance suggested that there was a tendency for patients under GA to suffer more PONV (RR: 2.12, 95%CI: 0.95, 4.73, I^2^ = 75.2%, *P* = 0.07); and the difference between SA and GA was noticeable in the subgroup of open repair (Table 3).

## Discussion

Inguinal hernia repair is one of the most common surgeries in the world [1]. But it’s still undetermined that which anesthesia should be used. In order to improve the safety and effect of the repair, many previous studies or reviews attempted to compare local anesthesia and regional anesthesia and general anesthesia, and to disclose the advantages and disadvantages among these anesthesia. Our meta-analysis of eleven studies comparing SA and GA showed that patients receiving SA might have less pain intensity post operatively and it seems that SA was associated with higher patient satisfaction than GA, which suggests that SA can be an effective option for pain relief in hernia repair compared to the gold standard GA. However, It can not be confirmed that SA is better than GA as reported by previous studies, because surgery time in the SA group was longer and patients receiving SA might have an increased risk of postoperative headache and urinary retention, especially following laparoscopic repair.

Surgery duration and operating time in SA group for laparoscopic inguinal hernia repair were longer as compared to GA group, which was in line with most previous studies; while we found comparable results between the two groups for open hernia repair. However, significant heterogeneity precluded us to draw a conclusive conclusion, and further limitations on these results might be small sample size and operator variability. More robust evidence therefore is needed to verify our findings. Moreover, the hospital stay period is an important parameter to explore the effectiveness of the techniques. By data synthesis, we concluded that patients treated by either SA or GA had a similar hospitalization.

Pain in the early postoperative period, after inguinal hernia operations, is the most common patient complaint. Our meta-analysis showed that pain scores at 4 h and 12 h post operatively were lower in SA group than GA group, no matter following laparoscopic or open repairs. That is, compared with GA, SA shows an advantage in term of early postoperative pain as demonstrated by many studies [18, 19]. Furthermore, we found the superiority of SA over GA in early postoperative pain was more significant in laparoscopic repair than open repair. Less early pain in the SA group can help patients to breathe easier and get mobilized earlier, and reduces the need for additional analgesia. Likewise, patient satisfaction increases in the SA group attributed to less pain during the first postoperative hours and the similar length of hospital stay [20]. All the eleven studies analyzed concluded that the patients under SA techniques had slightly or significantly better satisfaction when compared with GA, following either open hernia repair or laparoscopic hernia repair. Besides, through our meta-analysis of four cohorts we found patients seem to be more satisfied with SA for inguinal hernia repair, especially in laparoscopic method. This means patients were happy and would probably recommend SA to his friends. However, given the fact that the sample size in the open repair group was relatively small, the outcomes need to be interpreted with caution and similar studies based on surgical approaches under SA and GA are warranted.

General complications, as one of the most determinant outcome measures, were reported comparable between groups in our results, including scrotal edema, seroma, wound infection, recurrence, shoulder pain. Urinary retention was one of the most frequent postoperative complications. Contradiction regarding urinary retention always exists. A higher frequency of urinary retention was often reported in previous studies. By meta-analysis, we found it was a tendency for patients under SA to experience more urinary retention than GA, in agreement with the most recent guidelines concluding that urinary retention might be more frequent following regional anesthesia [21]. Moreover, Reiner and his colleagues found that the age of the patients with urinary retention was significantly higher than patients without urinary retention, and suggested that urinary retention is seen more often in elderly patients [22]. Another study demonstrated that using short-acting agent, lidocaine, for SA virtually eliminates problems with urinary retention that occurs with long-acting SA agents [3]. Therefore, a deeper search into the incidence of urinary retention among specific groups with large sample size and adequate data is needed.

To our knowledge, headache is a very common complication following SA that always draws our attention. Our analysis showed that the incidence of headache was indeed higher in patients under SA than GA, but this difference was not significant in open herniorrhaphy. It’s hypothesized that varying use of anesthetic may have an influence on this outcome in the open repair group; and insufficient data for subgroup analysis may be responsible for the inconsistency. PONV is another important postoperative adverse effect that discomforts patients [23]. From the pooled analysis, we detected a trend that PONV created a higher morbidity in the GA group, which reached agreement with most studies. PONV is highest after GA, especially when nitrous or opiates or reversal agents are utilized and has been reported in up to 60–70% of patients [24]. The incidence is as high as 30% even with the newer agents like propofol and isoflurane [25]. It seems that the type of anesthetic agents used in the surgery influence the frequency of PONV among patients under GA. But to our knowledge, in most cases, SA has an advantage over GA in terms of the incidence of PONV due to the freedom from endotracheal intubation.

Several limitations should be acknowledged. The major limitations were significant heterogeneity and small study sample size that will greatly weaken our conclusion. Moreover, it’s reported by a Danish study with a very large sample that the risk of complications after herniorrhaphy increased with increasing age [26]; patient age can be a critical factor affecting our results of complications, but subgroup analysis based on age did not work in our meta-analysis for the lack of information. Therefore, clinical application of our assessment regarding complications should be cautious. Another limitation of the analysis was the outcome measures failed to cover some other commonly concerned aspects due to inadequate data, such as minor complications, conversion to GA during repair, and return to work and normal activity. Furthermore, the combined analysis of RCTs and cohort studies produces less powerful evidence than the studies only recruiting RCTs. To improve the future comparison of inguinal hernia repair studies, a consensus should be reached regarding study designs and outcome measures.

## Conclusion

In conclusion, SA is a feasible and effective method for inguinal hernia repair in adults comparable to the gold standard GA as far as postoperative pain and patient satisfaction are concerned. However, patients receiving SA need a longer surgery time and may experience more urinary retention and headache postoperatively, especially following laparoscopic repair. Therefore, SA can be another good choice for pain relief in inguinal repair, but it can’t be confirmed that SA is better than GA. Further studies are still warranted to validate our conclusions.

## Data Availability

All data generated or analysed during this study are included in this published article.

## Abbreviations

CI: Confidence intervals
GA: General anesthesia
PONV: Postoperative nausea and vomiting
RCT: Randomized controlled trails
RR: Relative ratios
SA: Spinal anesthesia
SMD: Standardized mean difference
WMD: Weighted mean difference
